# Xiao-Yao-San, a Chinese Medicine Formula, Ameliorates Chronic Unpredictable Mild Stress Induced Polycystic Ovary in Rat

**DOI:** 10.3389/fphys.2017.00729

**Published:** 2017-09-22

**Authors:** Hao-Yu Sun, Quan Li, Yu-Ying Liu, Xiao-Hong Wei, Chun-Shui Pan, Jing-Yu Fan, Jing-Yan Han

**Affiliations:** ^1^Department of Integration of Chinese and Western Medicine, School of Basic Medical Sciences, Peking University Beijing, China; ^2^Tasly Microcirculation Research Center, Peking University Health Science Center Beijing, China; ^3^Key Laboratory of Microcirculation, State Administration of Traditional Chinese Medicine of the People's Republic of China Beijing, China; ^4^Key Laboratory of Stasis and Phlegm, State Administration of Traditional Chinese Medicine of the People's Republic of China Beijing, China; ^5^State Key Laboratory of Core Technology in Innovative Chinese Medicine Beijing, China

**Keywords:** chronic unpredictable stress, polycystic ovary, noradrenaline, beta 2 adrenergic receptor, granulosa cells autophagy

## Abstract

Chronic stress induces endocrine disturbance, which contributes to the development of polycystic ovary syndrome (PCOS), a condition that remains a challenge for clinicians to cope with. The present study investigated the effect of Xiao-Yao-San (XYS), a traditional Chinese medicine formula used for treatment of gynecological disease, on the chronic stress-induced polycystic ovary and its underlying mechanism. Female Sprague-Dwaley rats underwent a 3 weeks chronic unpredictable mild stress (CUMS) procedure to establish the PCOS model, followed by 4 weeks treatment with XYS (0.505 g/kg or 1.01 g/kg) by gavage. Granulosa cells were exposed to noradrenaline (1 mM) *in vitro* for 24 h, followed by incubation with or without XYS-treated rat serum for 24 h. Post-treatment with XYS ameliorated CUMS-induced irregular estrous cycles and follicles development abnormalities, decrease of estradiol and progesterone level as well as increase of luteinizing hormone in serum, reduced cystic follicles formation and the apoptosis and autophagy of granulosa cells, attenuated the increase in dopamine beta hydroxylase and c-fos level in locus coeruleus, the noradrenaline level in serum and ovarian tissue, and the expression of beta 2 adrenergic receptor in ovarian tissue. Besides, XYS alleviated the reduction of phosphorylation of ribosomal protein S6 kinase polypeptide I and protein kinase B, as well as the increase of microtubule-associated protein light chain 3-I to microtubule-associated protein light chain 3-II conversion both *in vivo* and *in vitro*. This study demonstrated XYS as a potential strategy for CUMS induced polycystic ovary, and suggested that the beneficial role of XYS was correlated with the regulation of the sympathetic nerve activity.

## Introduction

Polycystic ovary syndrome (PCOS) is a common endocrine disorders in women of reproductive age featured by hyperandrogenism, chronic anovulation, polycystic ovaries on ultrasonography and abnormalities of folliculogenesis (Salelic et al., 2004; Norman et al., 2007). In the etiology of PCOS, psychological stress has attracted increasing attention (El Hayek et al., 2016). The patients with PCOS commonly present a higher degree of emotional distress (Greiner et al., 2005; Veltman-Verhulst et al., 2012). On the other hand, elevated risk for psychiatric disorders is observed as well in the sisters and brothers of women with PCOS, suggesting that the associations between PCOS and psychiatric disorders are partly due to a common etiology, including shared genetic factors (Cesta et al., 2016). Previous studies have demonstrated that chronic stress elevates the activity of sympathetic nervous system, increases the release of noradrenaline (NE) and nerve growth factor in the ovary (Lara et al., 1993; Paredes et al., 1998; Dorfman et al., 2003), and induces cystic follicles formation through the activation of beta adrenergic receptor (Fernandois et al., 2012; Luna et al., 2012). Moreover, chronic stress affects the secretion of gonadotropin and sexual hormone via hypothalamus-pituitary-ovary axis (Toufexis et al., 2014), and subsequently leads to follicle development abnormalities (Wu et al., 2012). However, how stress causes the impairment of follicles remains unclear.

The survival and death of granulosa cells have been recognized as one of the critical factors that impact the fate of follicles (Matsuda et al., 2012). Apoptosis and autophagy are two forms of programmed cell death, and the latter form involves the process in which a double-membrane vesicle called autophagosome carries cytoplasmic material to the lysosome (Mizushima and Komatsu, 2011). It has been reported that both apoptosis and autophagy can be induced in granulosa cells and are involved in the control of follicles development (Choi et al., 2010, 2013), which is modulated by various signaling pathways including phosphoinositide3-kinase/protein kinase B (PI3K/Akt) and mammalian target of rapamycin (mTOR) pathways (Pyo et al., 2012). However, the study on the role of apoptosis and autophagy in stress-induced polycystic ovary is limited.

Xiao-Yao-San (XYS) is a traditional Chinese medicine formula widely used for treatment of gynecological diseases and depression. In addition, studies showed that XYS can regulate hypothalamus-pituitary function (Jiang et al., 2016), protect the hippocampal neuron (Liang et al., 2013; Meng et al., 2013) in chronic stress-induced anxiety model. However, the role of XYS in chronic stress-caused polycystic ovary has not been explored.

The present study aimed to investigate the ameliorative effect of XYS on chronic stress-induced polycystic ovary and follicle development abnormalities, and gain insight into its underlying mechanism.

## Materials and methods

### Animals

Female Sprague-Dawley rats weighing 220 ± 20 g, 8 weeks-old, were obtained from the Animal Center of Peking University Health Science Center with the certificate number SCXK 2006-0008. The animals were housed at 24 ± 1°C and relative humidity of 50 ± 1% under a 12 h light/dark cycle and given standard laboratory diet and water. The experimental procedures were in accordance with the recommendations of U.K. Animals (Scientific Procedures) Act, 1986 and associated guidelines, EU Directive 2010/63/EU for animal experiments. All animals were handled according to the guidelines of the Peking University Animal Research Committee. The experimental protocol was approved by the Committee on the Ethics of Animal Experiments of Peking University Health Science Center (LA2016314).

### Reagents

XYS granules was provided by Guangdong Yi Fang Pharmaceutical Co Ltd. (Guangzhou, China), which consists of *radix bupleuri* (chaihu) (16.2%), *angelica* (danggui) (16.2%), *radix paeoniae alba* (baishao) (16.2%), *rhizoma atractylodis macrocephalae* (baizhu) (16.2%), *poria* (fuling) (16.2%), *ginger* (shengjiang) (5.4%), *mint* (bohe) (5.4%), and *glycyrrhizae* (gancao) (8.1%). Antibodies against beta 2 adrenergic receptor (β2R), S6K I, phosphor-p70 S6K I (Thr229) and c-fos were purchased from Abcam (Cambridge, UK). Antibodies against Bcl-2 (B-cell lymphoma-2), Bax (B-cell lymphoma-2 associated X protein), cleaved caspase-3 (cleaved cysteinly aspartate specific proteinase-3), microtubule-associated protein light chain 3A (LC3A), LC3B, Akt and phosphor-Akt (Ser473) were purchased from Cell Signaling (Danvers, MA, USA). Antibody against dopamine beta hydroxylase (DβH) was purchased from Thermo Scientific (Rockford, IL, USA). (-)-Noradrenaline (NE) was from Sigma Chemical Co (St Louis, MO, USA). Pregnant mare serum gonadotropin was from ProSpec (Ness Ziona, Israel).

### Experiment protocols

Animals underwent a week of adaptive breeding and estrous cycle determination, rats with normal estrous cycle were enrolled and divided into the following 6 groups randomly: (1) Control group, (2) Control+XYS (1.01 g/kg) group, (3) CUMS group, (4) CUMS+saline (NS) group, (5) CUMS+XYS (0.505 g/kg) group, and (6) CUMS+XYS (1.01 g/kg) group. The number of animals for determination of each parameter in each group is detailed in Table 1.

**Table 1:** Number of animals for different experimental groups and various parameters.

	**Control**	**Control+XYS (1.01 g/kg)**	**CUMS**	**CUMS+NS**	**CUMS+XYS (0.505 g/kg)**	**CUMS+XYS (1.01 g/kg)**	**Total**
Observation of estrous cycle	11	11	11	11	11	11	66
HE staining	(3)		(3)	(3)	(3)	(3)	(15)
Immunohistology staning	(3)		(3)	(3)	(3)	(3)	(15)
NE in tissue	8		8	8	8	8	40
NE, estradiol, progesterone, luteinizing hormone in serum	(8)		(8)	(8)	(8)	(8)	(40)
western blot	(6)		(6)	(6)	(6)	(6)	(30)
Total	19	11	19	19	19	19	106

*The numbers in the brackets represent the number of the animals used in the respective experiment and these animals were among those denoted above by the numbers free of brackets*.

The dosage of XYS used in the experiments was calculated based on that for human, with 0.505 g/kg being equivalent to and 1.01 g/kg 2-fold the dose (27g crude drug/60 kg), respectively, used in clinic.

CUMS used in the present study consisted of 10 kinds of stressors: (1) 24 h food deprivation, (2) 24 h water deprivation with empty drinking bottles, (3) 24 h bedding deprivation, (4) 24 h wet bedding, (5) 24 h tilt cage (cages were tilted to 45° from the horizontal), (6) 2 min clamp tail (1 cm from the tail tip), (7) 2 h cold stress in low temperature incubator at 4°C, (8) 1 h shaker stress (140 rpm), (9) 5 min cold swim at 4–6°C, and (10) 3 h restraint. The animals in the CUMS group, CUMS+NS group, CUMS+XYS (0.505 g/kg) group and CUMS+XYS (1.01 g/kg) group were subjected to 3 weeks CUMS with two randomly selected stressors imposed on the animals every day. During the same period of time, the animals in Control group and Control+XYS (1.01 g/kg) group stood normally raised. In the week that followed, estrous cycle was determined for all animals, and only animals with disordered estrous cycle in CUMS groups were processed for treatment with or without XYS. XYS treatment started form the fifth week and lasted for 4 weeks, during this period, the animals in XYS treatment groups received XYS by gavage daily at a dose as indicated, while those in CUMS+NS group were given equivalent volume of NS in the same manner. XYS was suspended in NS before use. Estrous cycle was determined for each rat over the whole treatment period.

### Preparation of XYS containing serum

Female SD rats were administered by gavage with NS or XYS (1.01 g/kg) once a day for 3 days. 2 h after the final dose, the rats were sacrificed and blood was collected. The blood samples were centrifuged at 1,699 g for 15 min at 4°C, the serum was collected and incubated in a water bath at 56°C for 30 min for inactivation, and stored at −80°C before use (Cao et al., 2013).

### Granulosa cells (GCS) culture studies

Female SD rats postnatal 21 days were sacrificed by cervical dislocation under anesthesia and ovaries were excised 48 h after administration of pregnant mare serum gonadotropin (0.4 IU/g) by intraperitoneal injection. GCs were isolated by immersing the ovaries in 5% fetal bovine serum (FBS)-DMEM (dulbecco's modified eagle medium) and puncturing them with 26 gauge needles to release GCs (Abedini et al., 2016). GCs were seeded in 3.5 cm petri dish and cultured in 15% FBS-DMEM at 37°C with 5% CO_2_ for 48 h, followed by incubation in 15% FBS-DMEM with NE (1 mM) (NE group) or without NE (Control group) for 24 h. A proportion of cells in NE group were transferred to 15% control rat serum-DMEM (NE+NS group) or 15% XYS (1.01 g/kg) rat serum-DMEM (NE+XYS (1.01 g/kg) group) incubating for additional 24 h.

### Determination of estrous cycle

Determination of the estrous cycle was performed daily for a week before CUMS and 5 weeks after CUMS using vaginal smears observed under a light microscope (Marcondes et al., 2002).

### Histological evaluation of ovarian

Rats were anesthetized by 20% urethane (10 ml/kg) at the end of the experiment. Ovaries were excised, rinsed in NS, fixed in 4% paraformaldehyde in 0.1 M phosphate buffer solution (pH 7.4). 5 μm paraffin sections were prepared and stained by hematoxylin and eosin (HE). The follicles were classified according to their hisotological characteristics (Cruz et al., 2012) and the number of primordial, primary, secondary, antral, cystic, and atretic follicles was counted for each ovary in a total of 10 sections with a 50 μm interval between the successive two.

### Determination of NE, estradiol, progesterone and luteinizing hormone

At the end of the experiment, the rats were sacrificed and blood and ovaries were collected. The levels of NE in serum and ovarian tissue homogenate were determined by using ELISA kits (Eagle Biosciences, Nashua, NH, USA), and the levels of estradiol, progesterone and luteinizing hormone in serum were determined by using ELISA kits (R&D Systems, Minneapolis, MN, USA; Enzo Life Sciences, Farmingdale, NY, USA) according to the manufacturer's instructions.

### Terminal deoxynucleotidyl transferase-mediated dUTP-biotin nick end-labeling (TUNEL) staining

TUNEL staining was conducted for paraffin sections of rat ovary using an assay kit (Roche, Basel, Switzerland), according the manufactures instruction, and the nuclei were labeled with Hochest 33342 (Invitrogen, Camarillo, CA, USA). The number of the TUNEL positive cells in 5 fields was counted, and the average was calculated and expressed as cell number per field.

### Immunohistochemical staining

At the end of the experiment, the rats under anesthesia were infused via the left ventricle with 4% paraformaldehyde in 0.01 M phosphate buffer solution (pH 7.4), brain segments containing region of the locus coeruleus (bregma −9.80 to −10.04 mm) (Sabban et al., 2015) were removed and postfixed with the same fixative for 48 h, and immersed in 30% sucrose solution for at least 48 h at 4°C. The brain segments were cut into 10 μm sections with a cryostat microtome (CM1900; Leica, Nussloch, Germany). GCs were fixed with 4% paraformaldehyde in 0.1 M phosphate buffer solution for 20 min at the end of the experiment. The sections or cultured cells were permeabilized with 0.3% Triton X-100 for 30 min, then incubated with antibodies against β2R (1:50), LC3A (1:400), DβH (1:50) or c-fos (1:100) overnight at 4°C after blocked with goat serum, and then incubated with a fluorescent secondary antibody for 1.5 h at 37°C and Hochest 33342 (Invitrogen, Camarillo, CA, USA) for nuclei. Positive staining was examined by a laser scanning confocal microscope (TCS SP5, Leica, Mannheim, Germany).

### Western blotting assay

Rats under anesthesia were sacrificed at the end of the experiment, the ovarian tissue and brain tissue containing region of locus coeruleus were removed and immediately frozen at −80°C till use. GCs were collected following incubation sequentially with NE and control or XYS rat serum. GCs, ovarian and brain tissue were homogenized in lysis buffer containing the protease inhibitor. Proteins were separated by sodium dodecyl sulfate-polyacrylamide gel electrophoresis and transferred to polyvinylidene fluoride (PVDF) membrane. After non-specific binding sites were blocked with 5% bovine serum albumin in Tris-buffered saline Tween (TBS-T), PVDF membranes were incubated overnight at 4°C with the primary antibodies against β2R (1:1000), Bax (1:1000), Bcl-2 (1:1000), cleaved caspase-3 (1:500), S6K I (1:1000), phosphor-S6K I (Thr229, 1:1000), Akt (1:1000), phosphor-Akt (Ser473, 1:2000), LC3A (1:1000), LC3B (1:1000), and GAPDH (1:2000) in diluent buffer (3% bovine serum albumin and 0.1% TBS-T). After rinsing with TBS-T for 3 times, PVDF membranes were incubated with secondary antibody (1:3000, Cell Signaling Technology, Danvers, MA, USA) for 1 h at room temperature and washed by TBS-T for 3 times. Antibody binding was detected by enhanced chemiluminescence detection kit (APPLYGEN, Beijing, China). Bands were scanned and evaluated by Bio-Rad Quantity One software for quantification (Bio-Rad, Richmond, CA, USA). All the western blot experiments were repeated at least 3 times.

### Statistical analysis

All parameters were expressed as mean ± SE. Statistical analysis was performed using one-way ANOVA followed by Turkey test for multiple comparison. A probability less than 0.05 was considered to be statistically significant.

## Results

### XYS ameliorates rat estrous cycle dysfunction induced by CUMS

**Figure 1:**
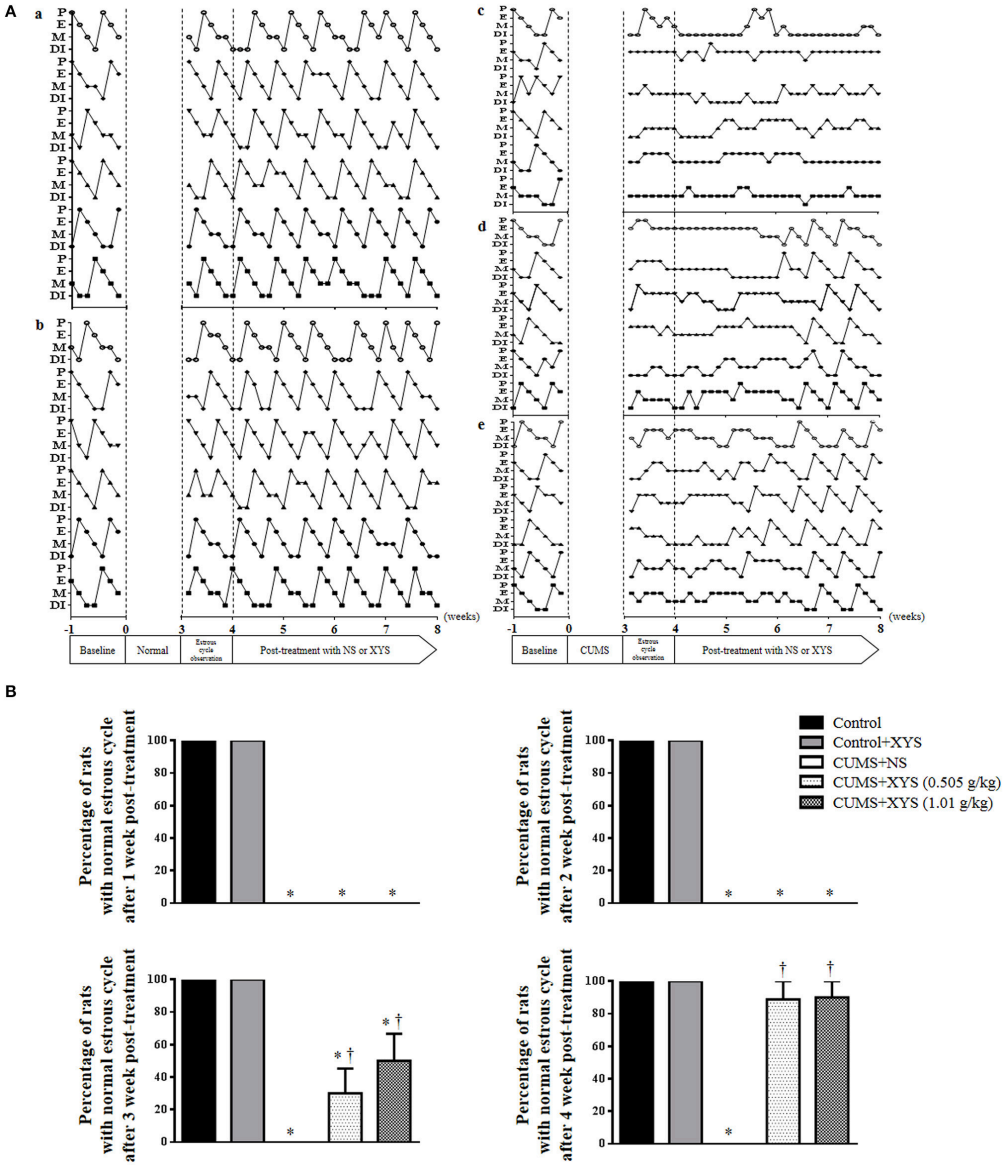
Effect of XYS post-treatment on rat estrous cycle. **(A)** Representative images exhibiting the changes of estrous cycle in Control **(a)**, Control+XYS (1.01 g/kg) **(b)**, CUMS+NS **(c)**, CUMS+XYS (0.505 g/kg) **(d)**, and CUMS+XYS (1.01 g/kg) **(e)** group, respectively. P, preoestrus; E, estrus; M, metaoestrus; DI, diestrus. **(B)** Statistical result of the percentage of rats with normal estrous cycle after 1, 2, 3, and 4 weeks XYS post-treatment. Results are presented as mean ± SE. ^*^P < 0.05 vs. Control group; ^†^*P* < 0.05 vs. CUMS+NS group, *n* = 11.

The estrous cycles of rats were assessed before and after CUMS. Figure 1A shows the representative profiles of estrous cycles in different groups. Rats of Control group (Figure 1Aa) and Control+XYS (1.01 g/kg) (Figure 1Ab) group revealed normal estrous cycles over the observation, while irregular estrous cycles were induced immediately after CUMS and persisted till the end of the experiment, if not interfered (Figure 1Ac). Post-treatment with XYS at 0.505 g/kg or 1.01 g/kg both recovered the irregular estrous cycles induced by CUMS starting from the third week of treatment (Figures 1Ad,e). Figure 1B is the quantification of the percentage of rats with normal estrous cycle during the post-treatment with XYS in different groups, which confirmed the results from the survey above.

### XYS alleviates rat polycystic ovary formation and follicles development abnormalities induced by CUMS

**Figure 2:**
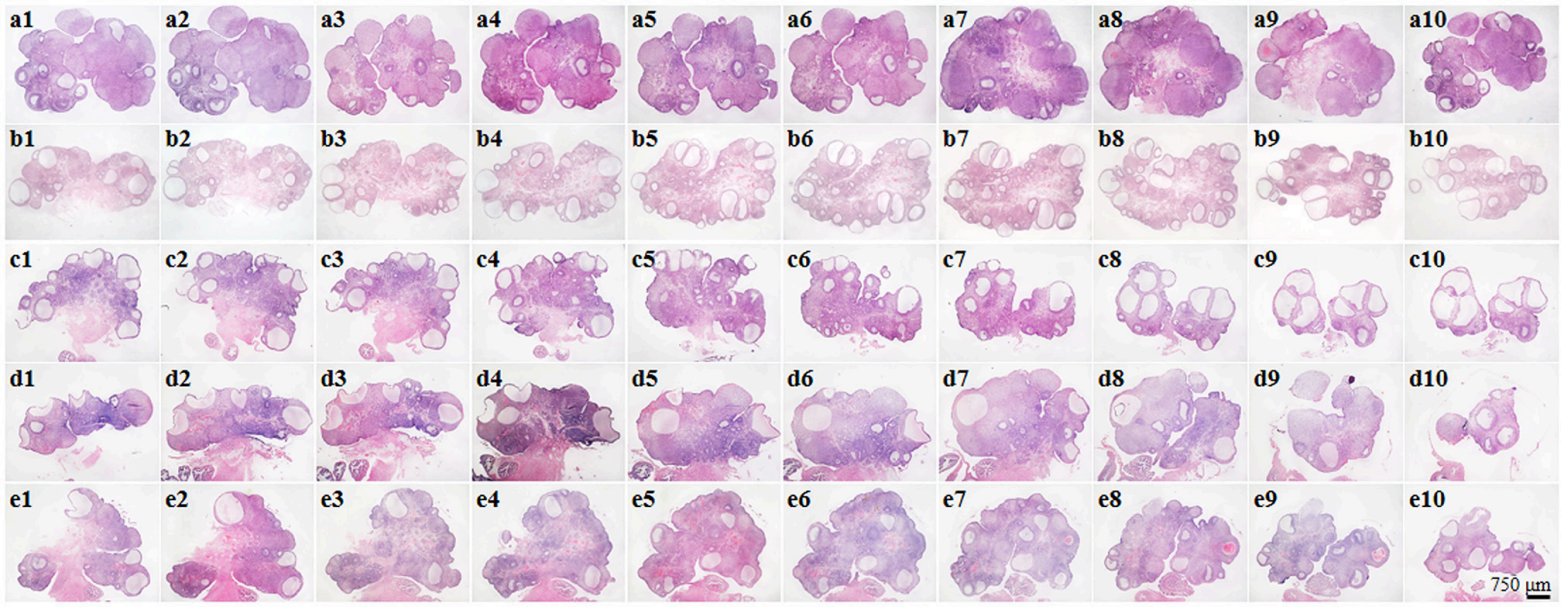
Effect of XYS post-treatment on histology of the ovarian tissue. Representative 10 sections of HE staining images of rat ovarian tissue in Control **(a1–a10)**, CUMS **(b1–b10)**, CUMS+NS **(c1–c10)**, CUMS+XYS (0.505 g/kg) **(d1–d10)**, and CUMS+XYS (1.01 g/kg) **(e1–e10)** group, respectively.

**Figure 3:**
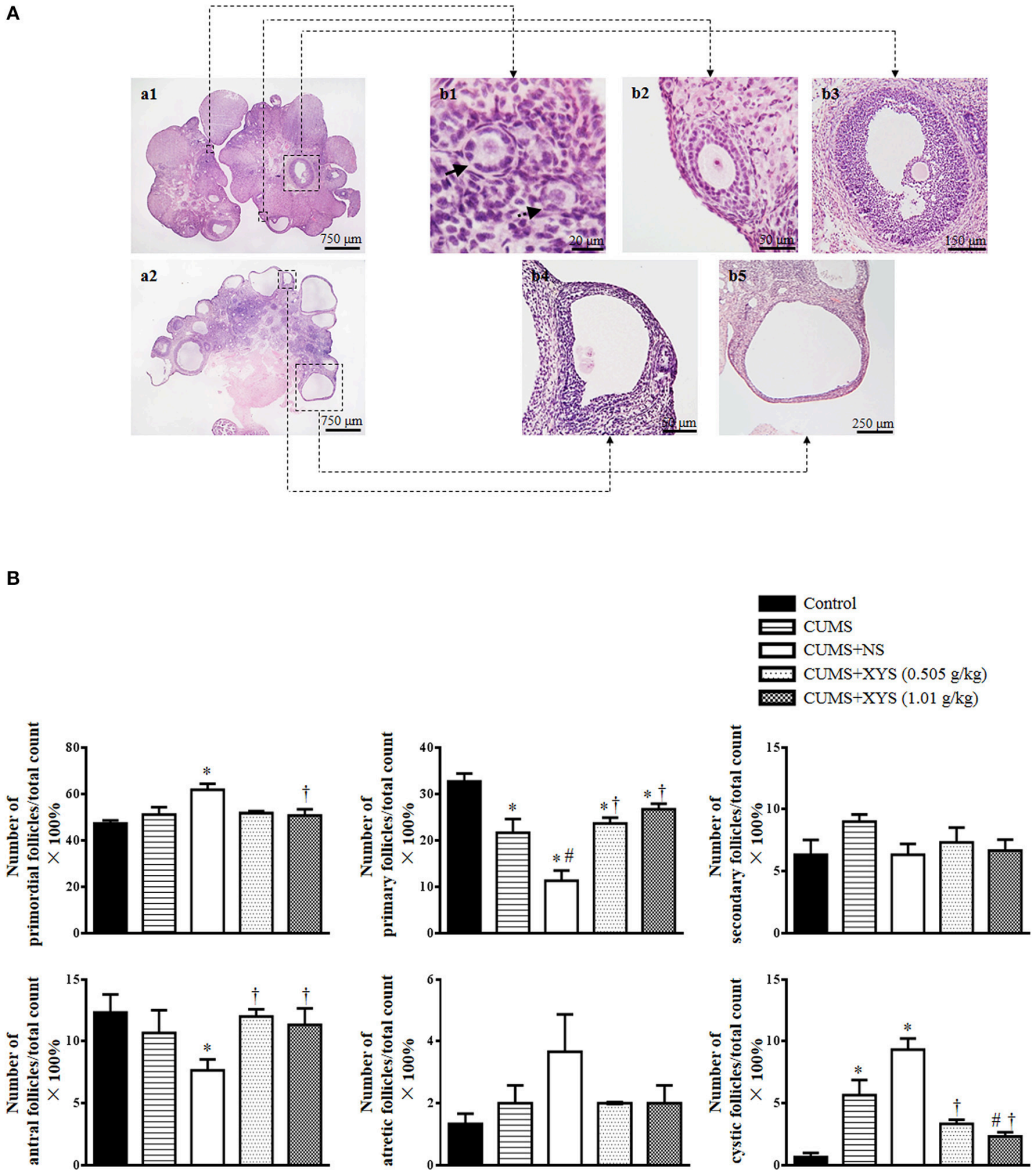
Effect of XYS post-treatment on follicle development of the ovary. **(A)** Representative HE staining images of rat ovarian tissue **(a1,a2)**. The area within the rectangle in each image in left panel is enlarged and presented right, exhibiting the representative images of primordial follicle (**b1**, dotted arrow), primary follicle (**b1**, solid arrow), secondary follicle **(b2)**, antral follicle **(b3)**, atretic follicle **(b4)**, and cystic follicle **(b5)**, respectively. **(B)** Statistical result of the percentage of the follicles in different development stages in various groups. Results are presented as mean ± SE. ^*^P < 0.05 vs. Control group; ^#^*P* < 0.05 vs. CUMS group; ^†^*P* < 0.05 vs. CUMS+NS group, *n* = 3.

Ovary morphology was examined by HE-staining and the representative images of different groups are presented in Figure 2. As compared to Control group (Figure 2a), numerous cystic follicles were observed in the ovaries of CUMS group (Figure 2b) and CUMS+NS group (Figure 2c). Noticeably, post-treatment with XYS (0.505 g/kg or 1.01 g/kg) significantly reduced the number of cystic follicles (Figures 2d,e). Illustrated in Figure 3A are the representative images of follicles at different developmental stages, and the percentage and number of the various follicles and corpus luteum are shown in Figure 3B and Table 2, respectively. At the end of CUMS, the number of both primary follicles and corpus luteum in the CUMS group was decreased, while the percentage of cystic follicles was increased. This tendency was further prominent 5 weeks after CUMS, accompanying with an increase in the percentage of primordial follicles and a decrease in the percentage of antral follicles. Post-treatment of XYS (0.505 g/kg and 1.01 g/kg) relieved the alternations mentioned above, suggesting the ameliorating effect of XYS on cystic follicles formation and follicle development abnormalities.

**Table 2:** Number and percentage of the follicles in different development stages and corpus luteum.

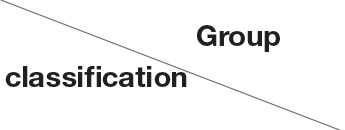	**Control**		**CUMS**		**CUMS**+**NS**		**CUMS**+**XYS (0.505 g/kg)**		**CUMS**+**XYS (1.01 g/kg)**	
	**Number**	**Percentage**	**Number**	**Percentage**	**Number**	**Percentage**	**Number**	**Percentage**	**Number**	**Percentage**
Primordial follicle	188 ± 11.53	47 ± 2	201.33 ± 28.38	51 ± 6	208.67 ± 31.66	61 ± 5^*^	176.67 ± 24.91	52 ± 1	194.33 ± 21.36	51 ± 4^†^
Primary follicle	131.33 ± 26.86	33 ± 3	84.67 ± 17.16^*^	22 ± 5^*^	38.67 ± 12.5^*^^#^	12 ± 4^*^^#^	81.67 ± 18.88^*^^†^	24 ± 2^*^^†^	102.33 ± 8.08^†^	27 ± 2^*^^†^
Secondary follicle	25 ± 9.54	6 ± 2	35.33 ± 3.51	9 ± 1	21.33 ± 6.03	6 ± 1	25 ± 5.57	7 ± 2	25.67 ± 7.51	7 ± 2
Antral follicle	46.33 ± 4.16	12 ± 3	42.33 ± 11.59	11 ± 3	26 ± 5.29^*^	8 ± 2^*^	42 ± 8.89	12 ± 1^†^	43.67 ± 8.33^†^	11 ± 3^†^
Cystic follicle	2.67 ± 1.53	0.7 ± 0.5	18.33 ± 13.32	6 ± 2^*^	31.33 ± 2.08^*^	9 ± 2^*^	11.67 ± 3.06^†^	3 ± 1^†^	8.33 ± 1.53^†^	2 ± 0.5^#^^†^
Atretic follicle	4.67 ± 2.89	1.2 ± 0.6	11.67 ± 7.51	2 ± 1	13 ± 7.81	4 ± 2	6.67 ± 1.53	2 ± 0.2	8 ± 4	2 ± 1
Total count	398 ± 42.23		393.67 ± 12.22		340.33 ± 36.3		343.67 ± 54.35		382.33 ± 24.91	
Corpus luteum	67.67 ± 9.61		38.67 ± 11.93^*^		20.33 ± 4.73^*^		48 ± 6.24^*^^†^		55.67 ± 7.02^†^	

Statistical result of the number and percentage of the follicles in different development stages and corpus luteum in various groups. Results are presented as mean ± SE.

* P < 0.05 vs. Control group;

# P < 0.05 vs. CUMS;

† *P < 0.05 vs. CUMS+NS group, n = 3*.

### XYS inhibits the increase of NE level in rat serum and ovarian tissue and ameliorates rat endocrine disorder induced by CUMS

**Figure 4:**
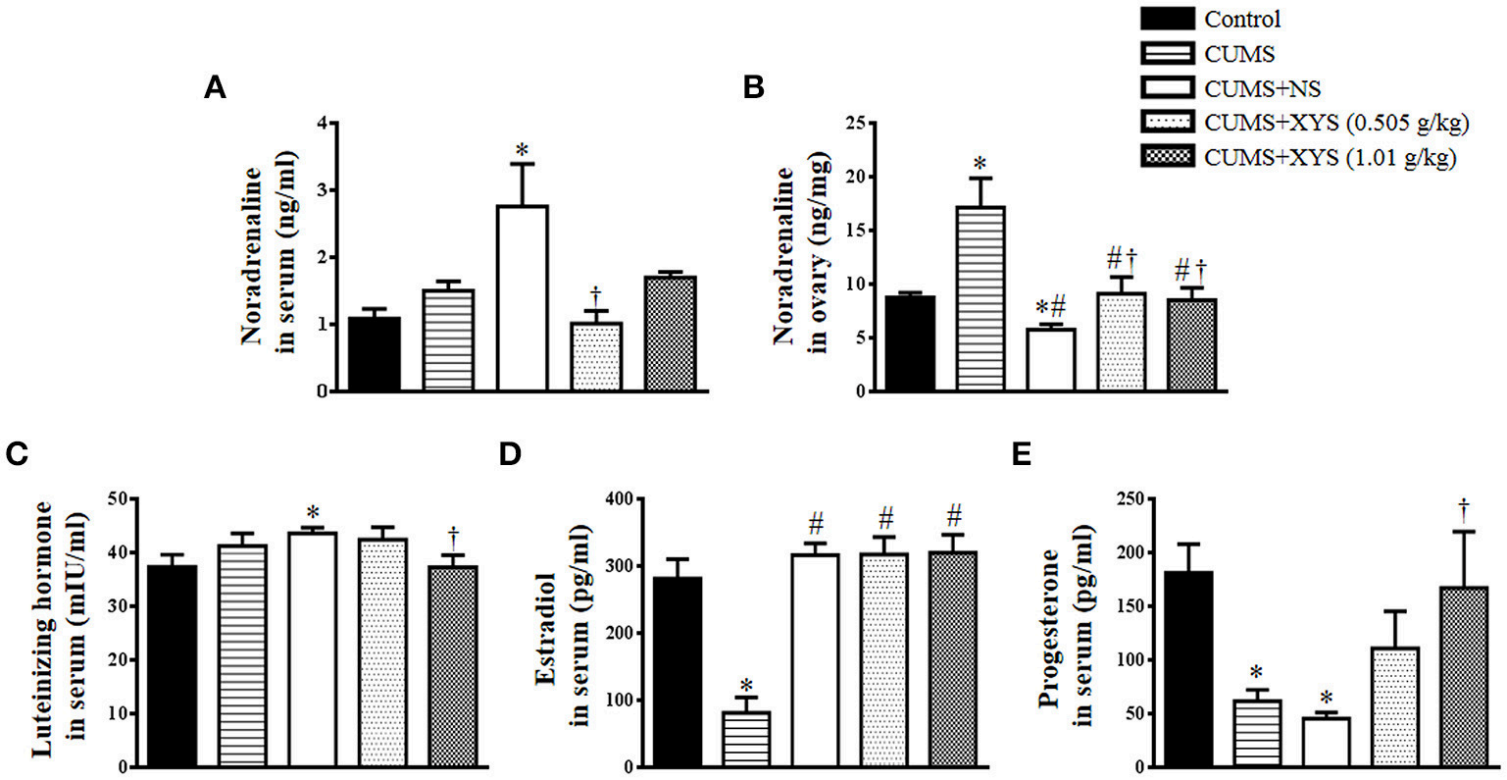
Effect of XYS post-treatment on the level of NE, luteinizing hormone, estradiol, and progesterone in rats. **(A)** NE concentration in serum in different groups. **(B)** NE concentration in ovarian tissue from different groups. **(C)** Luteinizing hormone in serum from different groups. **(D)** Estradiol in serum from different groups. **(E)** Progesterone in serum from different groups. Results are presented as mean±SE. ^*^P < 0.05 vs. Control group; ^#^*P* < 0.05 vs. CUMS group; ^†^*P* < 0.05 vs. CUMS+NS group, *n* = 8.

To examine the effect of XYS on the elevated activity of sympathetic nervous system induced by CUMS, NE level in rat serum and ovarian tissue was detected. Compared with Control group, the NE level in serum remained unchanged immediately after CUMS, but increased significantly at the end of the experiment (Figure 4A). On the other hand, NE level in ovarian tissue increased significantly after CUMS and decreased at the end of the experiment (Figure 4B). The alterations above were inhibited by post-treatment with XYS with significance.

Figures 4C–E present the results of the serum hormone level in different groups. Luteinizing hormone had no change immediately after CUMS challenge but increased at the end of the experiment if not interfered. This increase was protected by XYS treatment at 1.01 g/kg (Figure 4C). In contrast, the level of estradiol dramatically decreased after CUMS, but recovered completely at the end of the experiment, which was not affected by the treatment of XYS (Figure 4D). The level of progesterone in serum changed in response to CUMS challenge in a distinct manner in that it decreased markedly immediately after CUMS, which was persisted till the end of the experiment if not interfered, but prevented by treatment with XYS at 1.01 g/kg (Figure 4E).

### XYS relieves the increase of β2R in follicles after CUMS

**Figure 5:**
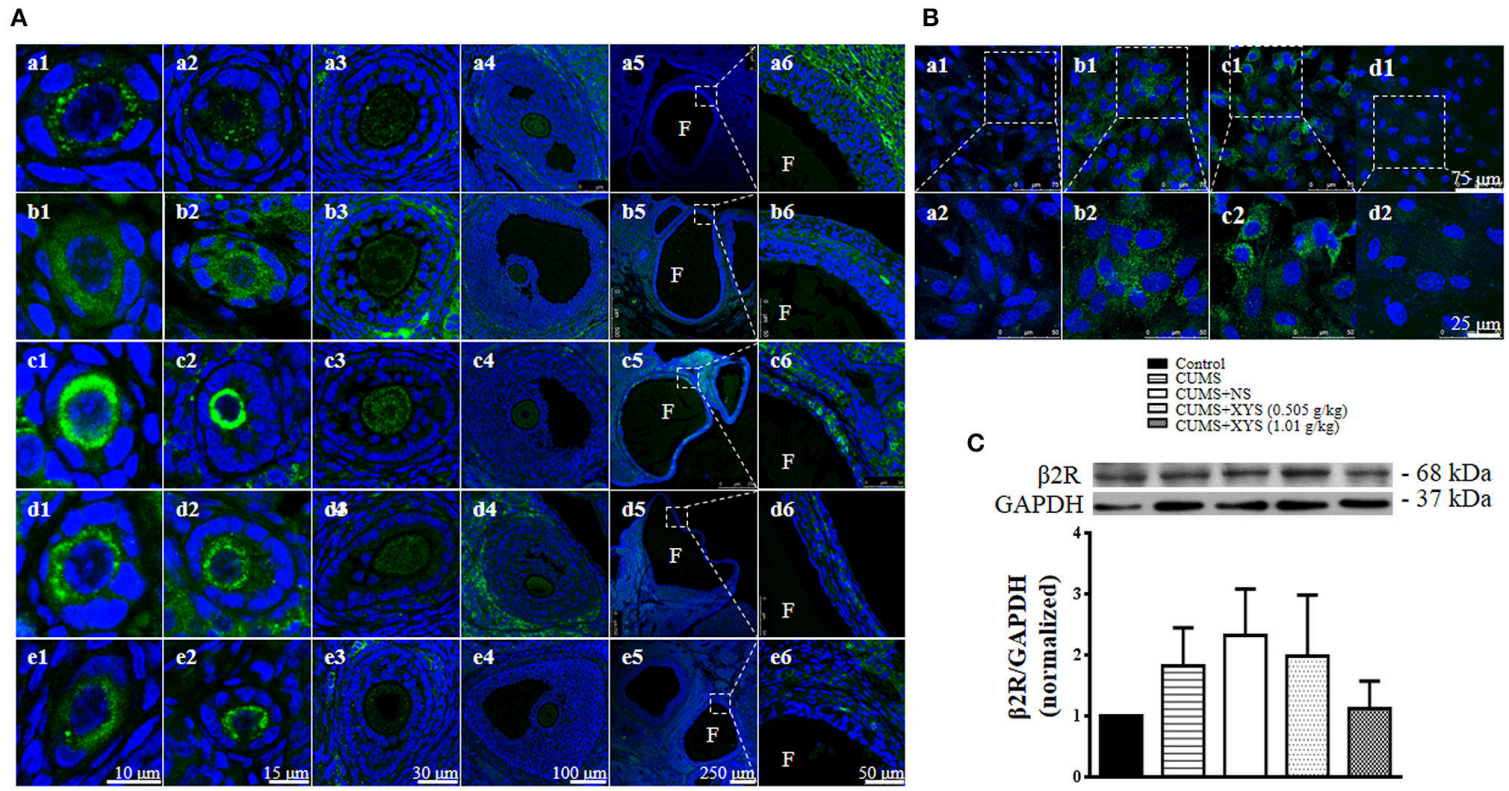
Effect of XYS post-treatment on the expression of β2R in ovarian tissue. **(A)** Representative immunofluorescence confocal images of primordial follicles (1, bar = 10 μm), primary follicles (2, bar = 15 μm), secondary follicles (3, bar = 30 μm), antral follicles (4, bar = 100 μm), cystic follicles (5, bar = 250 μm) and enlarged images of cystic follicles (6, bar = 50 μm) in Control **(a)**, CUMS **(b)**, CUMS+NS **(c)**, CUMS+XYS (0.505 g/kg) **(d)**, and CUMS+XYS (1.01 g/kg) **(e)** group, respectively. F, follicular antrum. The sections were immunochemically stained for β2R (green) and nuclei (blue). **(B)** Representative immunofluorescence confocal images of granulosa cells in Control **(a)**, NE **(b)**, NE+NS **(c)**, NE+XYS (1.01 g/kg) **(d)** group, respectively. The areas inside the rectangle of upper panel (1) are shown in the lower penal (2) at high magnification. The sections were immunochemically stained for β2R (green) and nuclei (blue). **(C)** Western blot for the expression of β2R in different groups with the quantification of β2R showing below. Results were presented as mean ± SE. No difference was noted among groups, *n* = 4.

To disclose the rationale behind the ameliorating effect of XYS on polycystic ovary formation and follicles development abnormalities, the expression of β2R in follicles was assessed by immunohistochemistry and western blot. Immunofluorescence staining (Figure 5A) shows that, compared with Control group, the expression of β2R increased in the primordial and primary follicles immediately after CUMS and further increased at the end of the experiment, which, however, was obviously relieved by post-treatment with XYS (Figures 5Aa1–e1,a2–e2). In accordance with the results in the ovarian tissue, NE exposure led to an increased expression of β2R in cultured granulosa cells (Figure 5B), which was attenuated by XYS. Western blot analysis (Figure 5C) shows a trend similar to immunofluorescence results, however there was no significant difference observed among the different groups.

### XYS inhibits the apoptosis of granulosa cells in the antral follicles induced by CUMS

**Figure 6:**
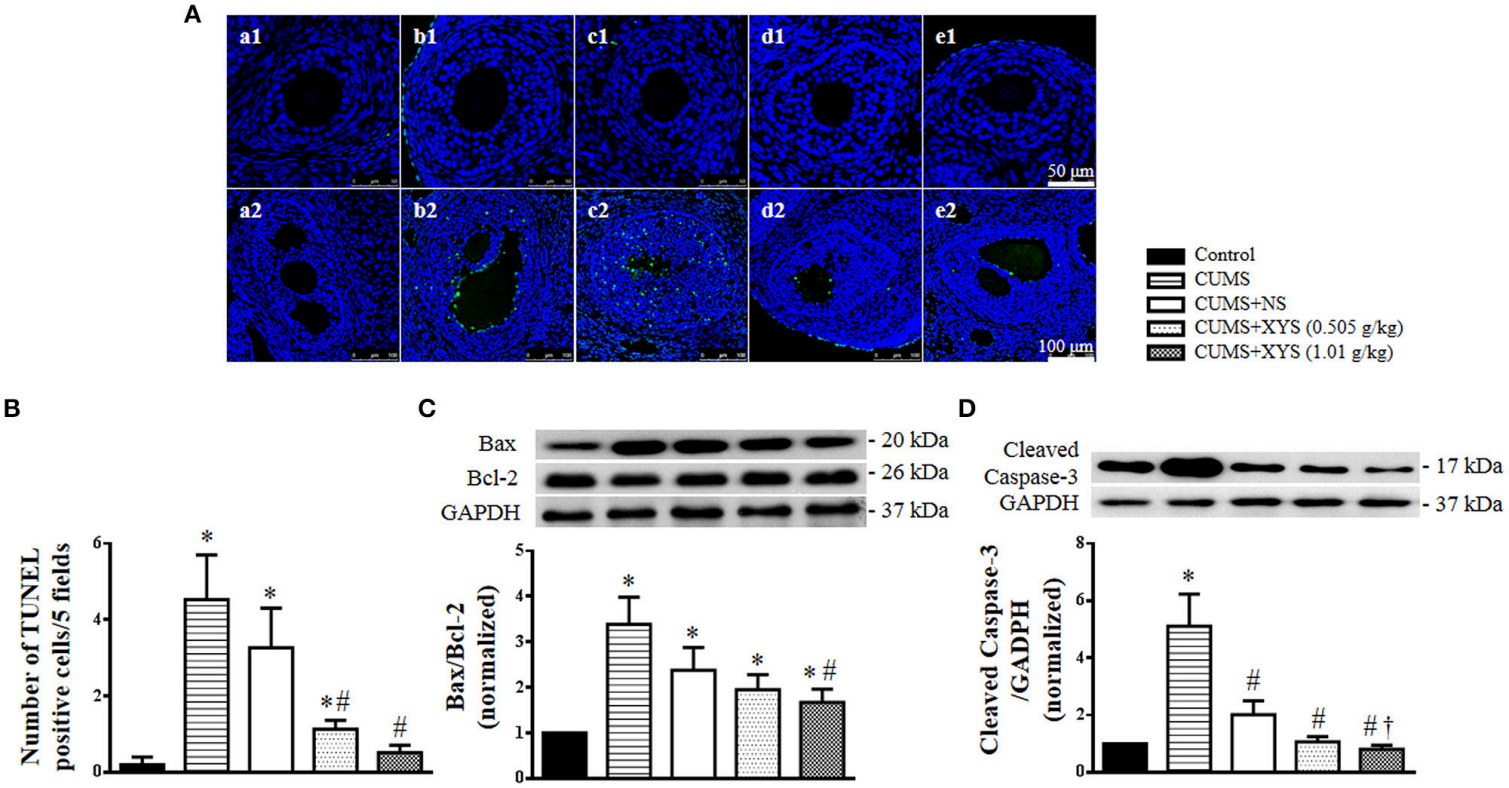
Effect of XYS post-treatment on the apoptosis of granulosa cells in ovarian tissue. **(A)** Representative TUNEL staining images of secondary follicles (1), and antral follicles (2) in Control **(a)**, CUMS **(b)**, CUMS+NS **(c)**, CUMS+XYS (0.505 g/kg) **(d)**, and CUMS+XYS (1.01 g/kg) **(e)** group, respectively. TUNEL positive cells are stained green. **(B)** Statistical result of number of TUNEL positive cells per 5 fields. **(C)** Western blot for the expression of Bax and Bcl-2 in different groups with the quantification of Bax/Bcl-2 showing below. **(D)** Western blot for the expression of cleaved caspase-3 in different groups with the quantification of cleaved caspase-3 showing below. Results are presented as mean ± SE. ^*^P < 0.05 vs. Control group; ^#^*P* < 0.05 vs. CUMS group; ^†^*P* < 0.05 vs. CUMS+NS group, *n* = 3 for TUNEL staining, *n* = 4 for western blot.

In order to investigate whether the formation of cystic follicles is associated with apoptosis of granulosa cells, we conducted TUNEL staining for ovarian tissues. Compared with the Control group, the antral follicles, but not the secondary follicles, from CUMS group demonstrated a high number of apoptotic granulosa cells (Figure 6A). On the other hand, less TUNEL positive cells were observed in the antral follicles from rats after XYS post-treatment. A quantitative assessment confirmed this result showing that the number of TUNEL positive cells significantly increased immediately after CUMS challenge, but decreased spontaneously at the end of the experiment, a process that was significantly accelerated by treatment with XYS (Figure 6B). In line with this result, Western blot analysis revealed that the ratio of Bax and Bcl-2 and the expression of cleaved caspase-3 varied among groups in a similar manner as that for TUNEL positive cells (Figures 6C,D).

### XYS inhibits the autophagy of granulosa cells in the antral and cystic follicles induced by CUMS

**Figure 7:**
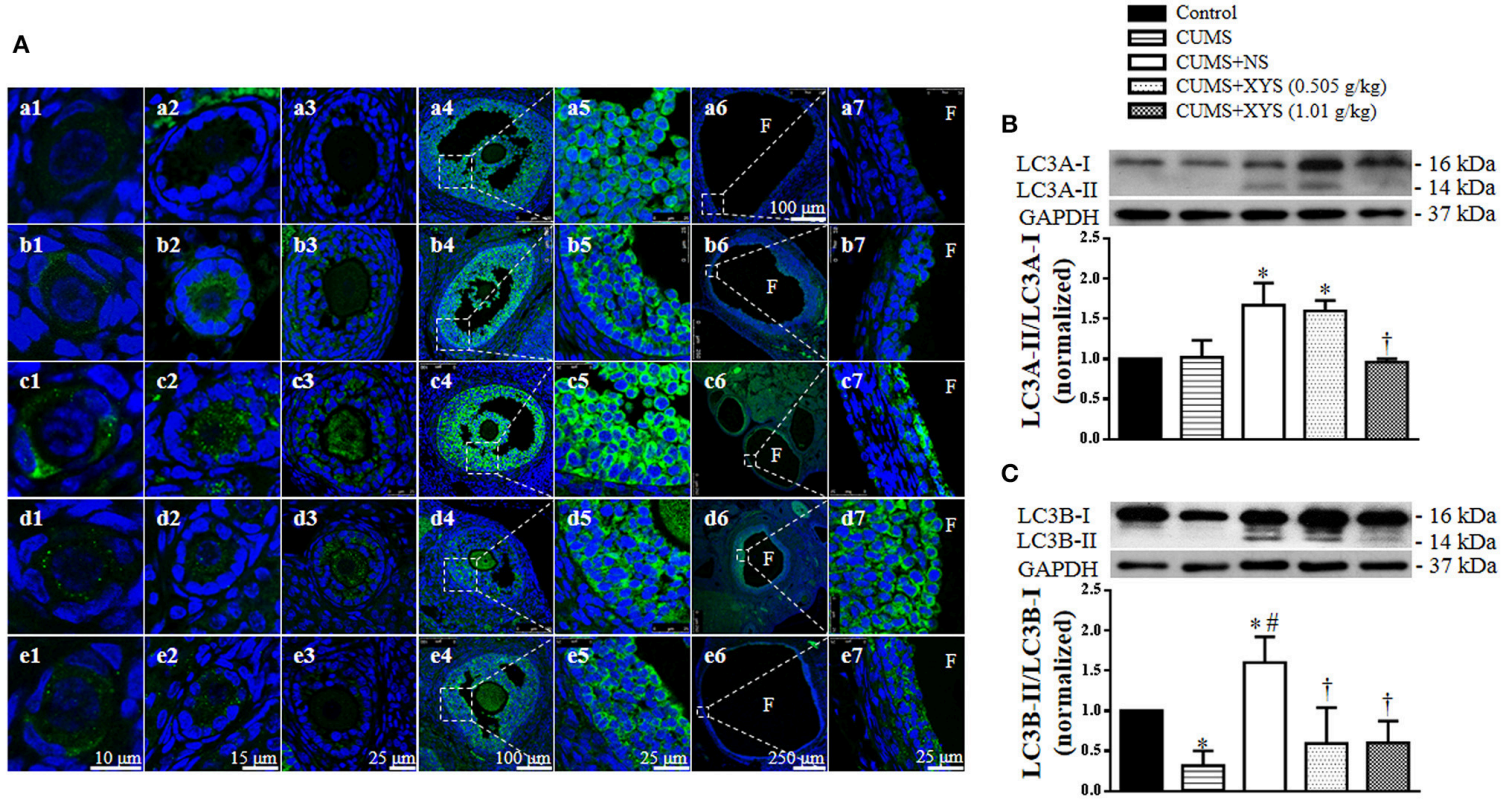
Effect of XYS post-treatment on the autophagy of granulosa cells in ovarian tissue. **(A)** Representative immunofluorescence confocal images of primordial follicles (1), primary follicles (2), secondary follicles (3), antral follicles (4), enlarged images of antral follicles (5), cystic follicles (6), and enlarged images of cystic follicles (7) in Control **(a)**, CUMS **(b)**, CUMS+NS **(c)**, CUMS+XYS (0.505 g/kg) **(d)**, and CUMS+XYS (1.01 g/kg) **(e)** group, respectively. F, follicular antrum. The sections were immunochemically stained for LC3A (green) and nuclei (blue). **(B)** Western blot for the expression of LC3A in different groups with the quantification of LC3A showing below. **(C)** Western blot for the expression of LC3B in different groups with the quantification of LC3B showing below. Results are presented as mean ± SE. ^*^P < 0.05 vs. Control group; ^#^*P* < 0.05 vs. CUMS group; ^†^*P* < 0.05 vs. CUMS+NS group, *n* = 4.

As an autophagy-related protein, LC3 is widely used as an autophagosome marker in mammalian cells (Klionsky et al., 2016). In view of the known involvement of autophagy in the development of follicles (Choi et al., 2010, 2013), we explored the expression of LC3 and the LC3-I to LC3-II conversion in the ovarian tissue by immunohistochemistry and Western blot. Compared with the Control group, the expression of LC3A in the granulosa cells of the antral and cystic follicles was elevated immediately after CUMS and at the end of the experiment, which was restored by treatment with XYS, however (Figure 7A). On the other hand, the conversion of LC3A-I to LC3A-II and LC3B-I to LC3B-II was increased as well at the end of the experiment, although this conversion did not change (LC3A-I to LC3A-II) or even decreased (LC3B-I to LC3B-II) immediately after CUMS. Nevertheless, the increased conversion of LC3 was inhibited significantly by post-treatment with XYS (Figures 7B,C).

### XYS inhibits the autophagy of granulosa cells induced by NE *in vitro*

**Figure 8:**
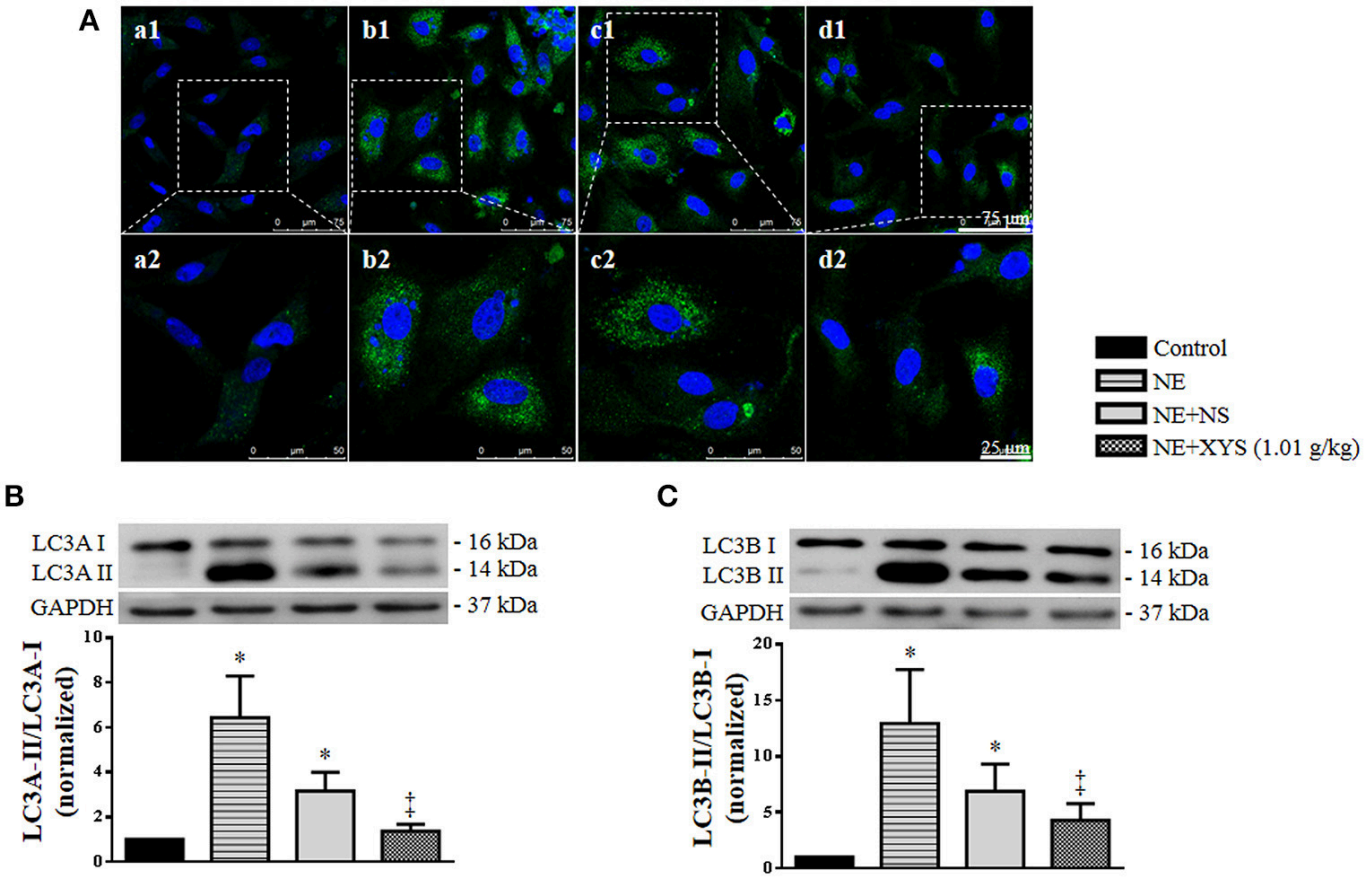
Effect of XYS post-treatment on the autophagy of cultured granulosa cells. **(A)** Representative immunofluorescence confocal images of granulosa cells in Control **(a)**, NE **(b)**, NE+NS **(c)**, and NE+XYS (1.01 g/kg) **(d)** group, respectively. The upper panel (1) is low magnification of the images, the areas inside the rectangle are shown in the lower penal (2) at high magnification. The sections were immunochemically stained for LC3A (green) and nuclei (blue). **(B)** Western blot for the expression of LC3A in different groups with the quantification of LC3A showing below. **(C)** Western blot for the expression of LC3B in different groups with the quantification of LC3B showing below. Results are presented as mean ± SE. ^*^P < 0.05 vs. Control group; ^‡^*P* < 0.05 vs. NE group, *n* = 4.

In order to explore whether the increased NE level in serum and ovarian tissue induced by CUMS contributes to the autophagy of granulosa cells and the protective effect of XYS, we examined the expression and localization of LC3 in granulosa cells *in vitro* by immunofluorescence staining. As shown in Figure 8A, NE evoked an increase in the expression of LC3 in the cytoplasm of granulosa cells as compared with Control group, which was relieved by post-treatment with rat serum containing XYS (1.01 g/kg). The results of western blot analysis also confirmed that NE caused an elevated conversion of LC3A-I to LC3A-II and LC3B-I to LC3B-II in cultured granulosa cells (Figures 8B,C), which was inhibited by post-treatment with rat serum containing XYS (1.01 g/kg).

### Akt/mTOR/S6K I pathway is involved in the alleviative effect of XYS on the autophagy of granulosa cells induced by CUMS *in vivo* and by NE *in vitro*

**Figure 9:**
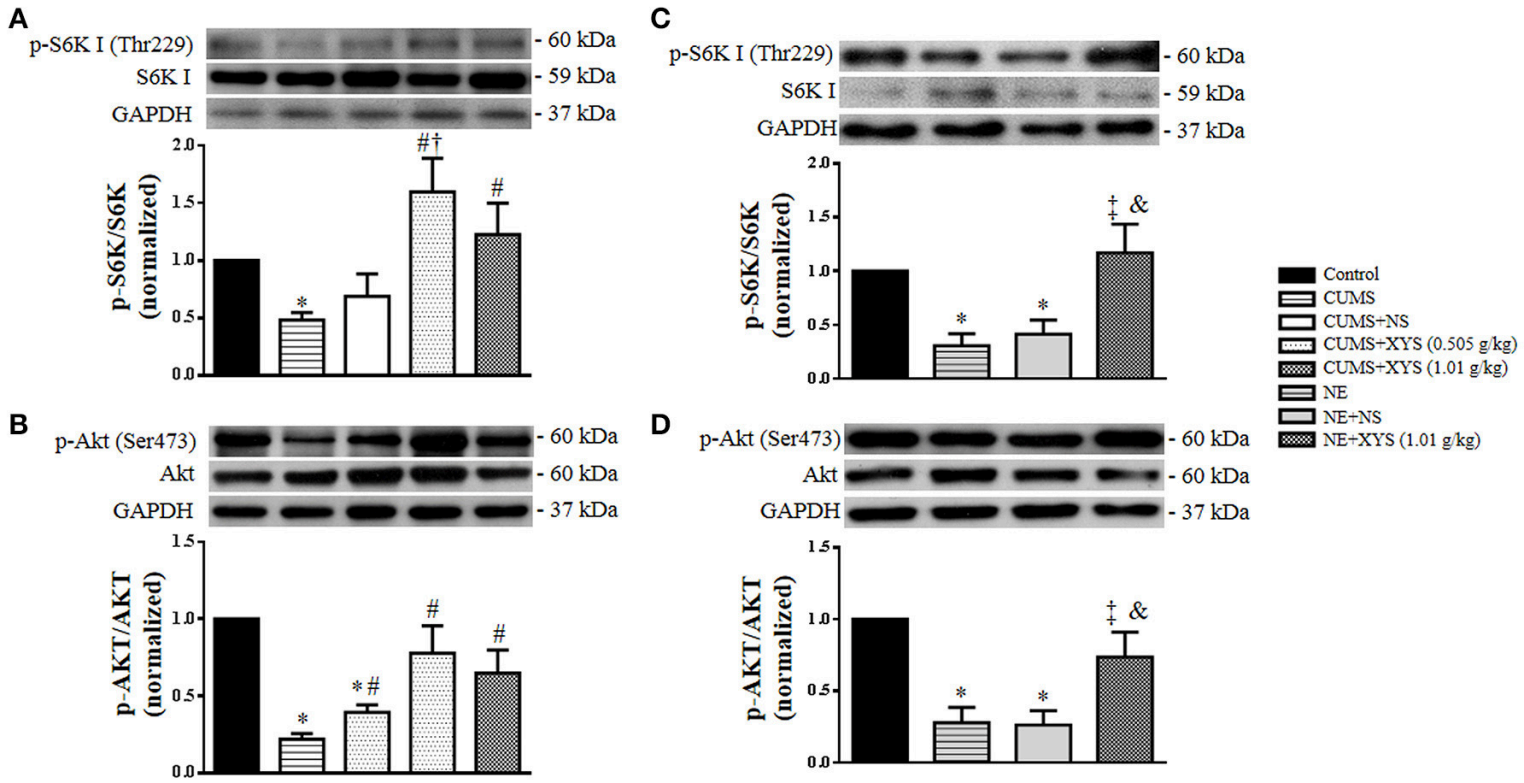
Effect of XYS post-treatment on the phosphorylation of S6K I and Akt in ovarian tissue and cultured granulosa cells. **(A)** Western blot for the phosphorylation of S6K I in ovarian tissue from different groups with the quantification of phosphorylation of S6K I showing below. **(B)** Western blot for the phosphorylation of Akt in ovarian tissue from different groups with the quantification of phosphorylation of Akt showing below. **(C)** Western blot for the phosphorylation of S6K I in cultured granulosa cells from different groups with the quantification of phosphorylation of S6K I showing below. **(D)** Western blot for the phosphorylation of Akt in cultured granulosa cells from different groups with the quantification of phosphorylation of Akt showing below. Results are presented as mean ± SE. ^*^P < 0.05 vs. Control group; ^#^*P* < 0.05 vs. CUMS group; ^†^*P* < 0.05 vs. CUMS+NS group; ^‡^*P* < 0.05 vs. NE group; ^&^*P* < 0.05 vs. NE+NS group, *n* = 4.

The mTOR pathway is known as a major suppressing signal of autophagy induction, which can be activated by PI3K/Akt signaling (Li et al., 2009; Qin et al., 2010). Moreover, both mTOR and PI3K/Akt pathway participate in the regulation of follicle development (Sobinoff et al., 2013). Thus, Western blot analysis was undertaken to assess the expression and phosphorylation of S6K I and Akt in ovarian tissues as well as in cultured granulosa cells from different groups. The results revealed that CUMS induced a decrease in phosphorylation of S6K I and Akt in ovarian tissues (Figures 9A,B), which was blunted significantly by post-treatment with XYS, suggesting the involvement of Akt/mTOR/S6K I pathway in attenuating effect of XYS on autophagy of ovarian tissues induced by CUMS. A similar result was observed *in vitro* in cultured granulosa cells exposed to NE (Figures 9C,D), hinting the key role of NE in CUMS challenge and the mechanism behind the effect of XYS.

### XYS reduces the increases level of DβH and c-fos in locus coeruleus

**Figure 10:**
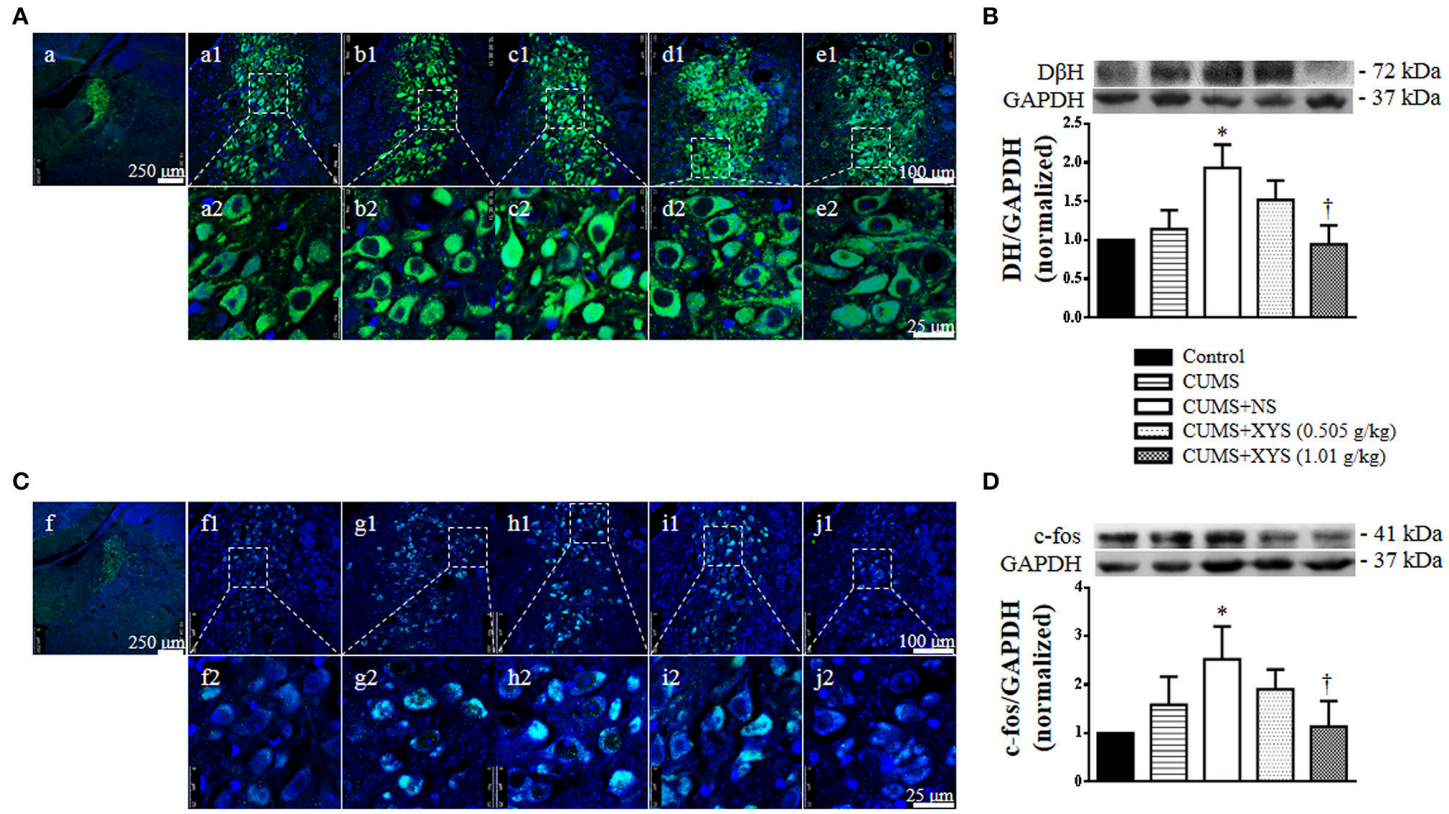
Effect of XYS post-treatment on expression of DβH and c-fos in locus coeruleus. **(A)** Representative immunofluorescence confocal images of locus coeruleus in Control **(a)**, CUMS **(b)**, CUMS+NS **(c)**, CUMS+XYS (0.505 g/kg) **(d)**, and CUMS+XYS (1.01 g/kg) **(e)** group, respectively. The upper panel (1) is low magnification of the images, the areas inside the rectangle are shown in the lower penal (2) at high magnification. The sections were immunochemically stained for DβH (green) and nuclei (blue). **(B)** Western blot for the expression of DβH in locus coeruleus of different groups with the quantification of DβH showing below. **(C)** Representative immunofluorescence confocal images of locus coeruleus in Control **(f)**, CUMS **(g)**, CUMS+NS **(h)**, CUMS+XYS (0.505 g/kg) **(i)**, and CUMS+XYS (1.01 g/kg) **(j)** group, respectively. The upper panel (1) is low magnification of the images, the areas inside the rectangle are shown in the lower penal (2) at high magnification. The sections were immunochemically stained for c-fos (green) and nuclei (blue). **(D)** Western blot for the expression of c-fos in locus coeruleus of different groups with the quantification of c-fos showing below. Results are presented as mean ± SE. ^*^P < 0.05 vs. Control group; ^†^*P* < 0.05 vs. CUMS+NS group, *n* = 4.

Since locus coeruleus is the principal site for brain synthesis of noradrenaline, we thus detected DβH, the rate-limiting enzyme of NE synthesis, and c-fos protein, a marker of neuron activity, in locus coeruleus by immunohistochemistry and Western bolt. As shown in Figure 10, the expression of DβH and c-fos in locus coeruleus increased significantly at the end of the experiment, which was protected by post-treatment of XYS (1.01 g/kg), indicating the potential of XYS to regulate the activity of sympathetic nervous system.

## Discussion

The present study demonstrated that CUMS caused follicle development abnormality, endocrine disturbance, irregular estrous cycles, increased apoptosis and autophagy in granulosa cells, along with an elevated NE level in plasma and ovary and activation of locus coeruleus. All the manifestations emerged in response to CUMS challenge were relieved by treatment with XYS, suggesting XYS as an option for treatment of follicles development abnormality in clinics, and highlighting the key role of NE in the mechanism behind the effect of XYS.

Follicle development abnormality, a condition that frequently occurs in polycystic ovary syndrome, accounts for a considerable proportion of female infertility. The detailed mechanism underlying follicle development disorder is not fully understood, however (Chang and Andersen, 2013). The present study found that CUMS successively induced follicle development abnormality in rat, providing an animal model of follicle development abnormality, which may contribute to understanding the mechanism of this abnormality and development of remedy to deal with it.

The granulosa cells are well recognized as a critical player in the development of follicles. The granulosa cells produce estradiol, insulin-like growth factor and other cytokines in the ovary, and express the receptors for hormones such as estradiol, follicle stimulating hormone, luteinizing hormone (Juengel et al., 2006), which participate in the regulation of follicle development. It is thus anticipated that any impairment of granulosa cells will results in disordered follicle development. Consistent with this notion, we observed an increased apoptosis and autophagy in the granulosa cells after CUMS stimulation, accompanying with a decreased level of estradiol and progesterone in plasma, indicating that granulosa cells were impaired by CUMS. Importantly, XYS restored these disorders meanwhile attenuated follicle development abnormality after CUMS, highly suggesting a likely implication of granulosa cells in the improving effect of XYS on aberrant follicles.

Previous studies demonstrated that chronic stress elevated the sympathetic nerve activity, increased the synthesis and release of NE in the ovary (Lara et al., 1993; Paredes et al., 1998; Dorfman et al., 2003), and impacted the follicle development and hormone secretion (Acuna et al., 2009; Luna et al., 2012). Moreover, beta adrenergic receptor agonist isoproterenol has been reported to cause polycystic ovary in adult rats, and the beta-blocker propranolol can prevent the formation of cystic follicles induced by isoproterenol and ameliorate the follicle development and hormone secretion (Fernandois et al., 2012). The results of the present study are consistent with the results above, and provide some further detail of the dynamic change of NE and beta adrenergic receptor in ovary in response to stress. We found that NE in ovary increased immediately after CUMS but returned to normal at the end of the experiment, while NE in plasma remained unchanged immediately after CUMS but increased significantly at the end of the experiment. These results imply that NE increased in ovary in response to the stress and then released to plasma. Interestingly, it appears that β2R in ovary increased immediately after CUMS and further increased over time. Taken together, these results suggest that although the CUMS-increased NE concentration in ovary returned to normal over time, the responsiveness of ovary to NE increased due to the increase in NE in plasma and the expression of β2R in ovary. Nevertheless, the increase in both plasma NE and ovary β2R was protected by XYS treatment. There are at least two ways in which XYS may interfere in the effect of NE on ovary. One is to affect ovary directly to intervene the interaction between NE and its receptors or the downstream signaling, and the other is to affect the noradrenergic neurons that innervate ovary.

In the present study, the first possibility was tested by using cultured granulosa cells, in view of the critical importance of these cells in regulation of follicle development and occurrence of programmed cell death in response to CUMS observed in the *in vivo* experiment. The results showed that exposure to NE led to not only an increased autophagy in cultured granulosa cells but also a decreased activation of AKT and S6K I, the pathways that are known to regulate negatively the autophagy and apoptosis (Levine and Kroemer, 2008). Importantly, both the increase in autophagy and decrease in activation of AKT and S6K I after NE exposure were restored by XYS, suggesting a direct impact of XYS on follicles by targeting AKT and S6K I pathways.

We next tested the likely involvement of locus coeruleus in the effect of XYS on follicle development disorder after CUMS. Locus coeruleus is a cluster of noradrenergic neurons in the brain, which mediates the responses to stress and the function of hypothalamus-pituitary (Szabadi, 2013). Particularly, locus coeruleus is synaptically connected to the preganglionic cell bodies of the ovarian sympathetic pathway (Marcelo et al., 2008). As expected, CUMS caused activation of locus coeruleus, as indicated by the increased expression of DβH and c-fos. XYS treatment protected the activation of locus coeruleus by CUMS, suggesting locus coeruleus as one of the targets that XYS acts to attenuate the stress-elicited elevation of NE and the resultant follicle development disorders.

Nevertheless, this study has some limitations. Firstly, we confirmed the key role of NE in triggering follicle development disorders and demonstrated the potential of XYS to counteract the NE effect by targeting both granulosa cells and locus coeruleus. However, the exact mechanism there by XYS exerts effect needs to be clarified by further studies, particularly for its effect on locus coeruleus. Secondly, XYS is a compound Chinese medicine containing multiple components. More studies are required to identify the component (s) responsible for the effects observed. Finally, follicle development is a complex process that is regulated by a spectrum of hormones, which are produced in different type of cells in follicles including granulosa cells, theca cells, and oocyte. The present study focused on the role of granulosa cells in initiation of follicle development disorder and the effect of XYS on this process. Whether other type of cells of follicles are implicated in the events concerned is not clear at present.

In conclusion, this study demonstrated that XYS ameliorated CUMS-induced follicle development abnormalities, and the inhibition of elevated NE release and β2R expression contributes to the therapeutic effect of XYS.

## Author contributions

HS performed the research, analyzed the data and wrote the manuscript; QL and YL contributed to animal experiments; XW contributed to immunochemistry analysis and cell culture; CP contributes to other experiments. JF and JH revised the manuscript; JH designed and funded the research, interpreted the data and finally approved the submission of this manuscript. All authors have read and agreed with the manuscript.

### Conflict of interest statement

The authors declare that the research was conducted in the absence of any commercial or financial relationships that could be construed as a potential conflict of interest. The reviewer SH and handling Editor declared their shared affiliation.

## Abbreviations

Akt: protein kinase B
Bax: B-cell lymphoma-2 associated X protein
Bcl-2: B-cell lymphoma-2
β2R: beta 2 adrenergic receptor
cleaved caspase-3: cleaved cysteinly aspartate specific proteinase-3
CUMS: chronic unpredictable mild stress
DβH: dopamine beta hydroxylase
DMEM: dulbecco's modified eagle medium
FBS: fetal bovine serum
GCs: granulosa cells
HE: hematoxylin and eosin
LC3: microtubule-associated protein light chain 3
mTOR: mammalian target of rapamycin
NE: noradrenaline
PCOS: polycystic ovary syndrome
PI3K: phosphoinositide3-kinase
PVDF: polyvinylidene fluoride
S6K I: ribosomal protein S6 kinase polypeptide I
TBS-T: Tris-buffered saline Tween
TUNEL: terminal deoxynucleotidyl transferase-mediated dUTP-biotin nick end-labeling
XYS: Xiao-Yao-San.
